# Physiological and morphological plasticity in response to nitrogen availability of a yeast widely distributed in the open ocean

**DOI:** 10.1093/femsec/fiae053

**Published:** 2024-04-10

**Authors:** Poppy Diver, Ben A Ward, Michael Cunliffe

**Affiliations:** Marine Biological Association, The Laboratory, Citadel Hill, Plymouth, PL1 2PB, United Kingdom; School of Ocean and Earth Science, University of Southampton, Waterfront Campus, European Way, Southampton, SO14 3ZH, United Kingdom; School of Ocean and Earth Science, University of Southampton, Waterfront Campus, European Way, Southampton, SO14 3ZH, United Kingdom; Marine Biological Association, The Laboratory, Citadel Hill, Plymouth, PL1 2PB, United Kingdom; School of Biological and Marine Sciences, University of Plymouth, Drake Circus, Plymouth, PL4 8AA, United Kingdom

**Keywords:** functional traits, marine fungi, marine yeast, nitrogen, plankton ecology

## Abstract

Yeasts are prevalent in the open ocean, yet we have limited understanding of their ecophysiological adaptations, including their response to nitrogen availability, which can have a major role in determining the ecological potential of other planktonic microbes. In this study, we characterized the nitrogen uptake capabilities and growth responses of marine-occurring yeasts. Yeast isolates from the North Atlantic Ocean were screened for growth on diverse nitrogen substrates, and across a concentration gradient of three environmentally relevant nitrogen substrates: nitrate, ammonium, and urea. Three strains grew with enriched nitrate while two did not, demonstrating that nitrate utilization is present but not universal in marine yeasts, consistent with existing knowledge of nonmarine yeast strains. *Naganishia diffluens* MBA_F0213 modified the key functional trait of cell size in response to nitrogen concentration, suggesting yeast cell morphology changes along chemical gradients in the marine environment. Meta-analysis of the reference DNA barcode in public databases revealed that the genus *Naganishia* has a global ocean distribution, strengthening the environmental applicability of the culture-based observations. This study provides novel quantitative understanding of the ecophysiological and morphological responses of marine-derived yeasts to variable nitrogen availability *in vitro*, providing insight into the functional ecology of yeasts within pelagic open ocean environments.

## Teaser

Assessment of the responses of marine-occurring yeasts to nitrogen availability suggests ecological plasticity.

## Introduction

Marine yeasts are a group of heterotrophic microbial eukaryotes active throughout diverse marine environments including the open ocean (Grossart et al. 2019). The term yeast unites fungi capable of unicellular growth, which occur exclusively within the phyla Ascomycota and Basidiomycota, but do not form a monophyletic group (El Baidouri et al. 2021). Environmental DNA-based surveys show that fungal community composition varies between marine ecosystems (Amend et al. 2019). Open ocean fungal communities are dominated by the Ascomycota and Basidiomycota at the surface (Wang et al. 2014) and in the deep ocean (Bass et al. 2007). The Chytridiomycota show high relative contributions in coastal waters (Taylor and Cunliffe 2016, Debeljak and Baltar 2023), in association with sea ice in the Arctic Ocean (Hassett and Gradinger 2016), and with low salinity in the Baltic Sea (Rojas-Jimenez et al. 2019). Early diverging groups also represent a major fraction of fungal diversity in coastal sediments (Picard 2017) and deep-sea sediments (Nagahama et al. 2011). Despite rich morphological and taxonomic diversity across marine ecosystems, yeasts dominate planktonic fungal diversity at the global scale (Hassett et al. 2020).

Yeasts are saprotrophs, releasing extracellular enzymes to break down high molecular weight organic matter into smaller compounds, which can then be taken up by osmotrophy, a nutritional strategy shared with marine bacteria (Worden et al. 2015). Cunliffe et al. (2017) identified active saprotrophs of phytoplankton-derived organic carbon in a coastal ecosystem, including *Malassezia* (Basidiomycota), which contains unicellular yeast species found throughout pelagic marine environments (Amend 2014, Boekhout et al. 2022). In addition to saprotrophy, marine yeasts are associated with other trophic interactions, including predation by zooplankton (Cleary et al. 2016), and parasitism of phytoplankton (Li et al. 2016) and zooplankton (Seki and Fulton 1969). Marine yeast abundances have been shown to fluctuate by several orders of magnitude over relatively short time-scales during a spring phytoplankton bloom (Priest et al. 2021), while interannual recurrence patterns of yeast-containing taxa further suggest a dynamic role for yeasts in marine plankton communities (Taylor and Cunliffe 2016, Chrismas et al. 2023). Absolute abundances of marine yeast taxa have been significantly positively correlated with environmental concentrations of nitrogen-containing compounds (Taylor and Cunliffe 2016, Priest et al. 2021), but quantitative data describing marine yeast responses to nitrogen availability are currently lacking in contrast with other major plankton groups.

Phytoplankton ecophysiology and community composition are driven by the concentration and chemical forms of nitrogen available (Glibert et al. 2016). Large diatoms dominate productive, nitrate-rich surface waters (e.g. Kudela and Dugdale 2000), whereas smaller phytoplankton such as *Cyanobacteria* dominate when reduced forms including ammonium and urea are the primary nitrogen sources (e.g. Berg et al. 2003). Despite strong links to seasonal bloom events, nitrate utilization is not a universal trait in marine phytoplankton, e.g. some ecotypes of the globally abundant picocyanobacterium *Prochlorococcus* lack the capacity for nitrate uptake (Moore et al. 2002). Nitrate assimilation is also restricted to a subset of yeast genera not including the model fission yeast genus *Schizosaccharomyces* (Siverio 2002), which had the highest relative sequence abundance of identified fungal genera across most oceanographic regions in a global metagenomic dataset (Hassett et al. 2020). In the marine water column, nitrate utilization could offer some yeasts a distinct advantage, especially when high-nitrate conditions trigger rapid diatom growth, providing ready supplies of both nitrogen and a suitable diatom-derived organic carbon substrate (Cunliffe et al. 2017). Efficient growth on ammonium and/or urea could sustain marine yeasts that are (a) unable to take up nitrate, or (b) in low-nitrate conditions, where microbial remineralization of reduced nitrogen fuels microbial production (Dugdale and Goering 1967, Glibert et al. 2016). To date, it has been difficult to address ecological hypotheses regarding marine yeast nitrogen dynamics, due to insufficient knowledge of their nitrogen uptake capabilities, specifically which nitrogen substrates can be used and whether nitrogen limits yeast growth at concentrations relevant to pelagic marine environments.

Nitrate (NO_3_^−^) is typically the most abundant form of fixed nitrogen in the ocean and nitrate concentration increases with depth in near-surface waters (Gruber 2008). Ammonium (NH_4_^+^) tends to be present at much lower apparent concentrations due to rapid microbial cycling in the water column (Glibert and Goldman 1981). Across the Atlantic Ocean, euphotic zone nitrate fluctuates by several orders of magnitude (0.005–33 µmol l^−1^), while ammonium is much less variable (0.109 ± 0.150 µmol l^−1^, maximum 5.2 µmol l^−1^) (Rees et al. 2006). Urea concentrations in the euphotic Atlantic are typically 0.15–0.35 µmol l^−1^, sometimes reaching 1.72 µmol l^−1^ (Painter et al. 2008), representing an important source of dissolved nitrogen for phytoplankton, particularly *Prochlorococcus* in the subtropical North Atlantic (Casey et al. 2007, Painter et al. 2008).

Phytoplankton nutrient utilization traits exhibit allometric scaling relationships across species and across major taxonomic groups, e.g. biomass-scaled nutrient affinities tend to decrease with increasing cell volume (Edwards et al. 2012). As cell size constrains microbial physiology and microbe–microbe interactions, it is considered a key functional trait regulating marine plankton biogeography (Barton et al. 2013). Allometric relationships have been investigated among filamentous marine fungi (Fuentes et al. 2015, Aguilar-Trigueros et al. 2017) and between major cell types of marine fungi (Thomas et al. 2022), but trait variations across marine yeast taxa, or within taxa under changing conditions are not well-documented. Two Pacific Ocean yeast isolates showed variable growth rates and cell volumes along a salinity gradient and the responses were strain-specific (Hernandez-Saavedra et al. 1995). Ecophysiological and morphological trait data covering a wider range of taxa and environmental conditions are needed to explore possible size-scaling relationships in marine yeasts and more broadly how yeast traits relate to their ecological function within marine plankton communities.

Culture-based approaches reveal that marine-derived yeast growth responds to abiotic environmental parameters such as temperature, salinity, and pH (Norkrans 1966, Hernandez-Saavedra et al. 1995, Breyer et al. 2023) and that carbon substrate preferences vary between marine fungal taxa (Thomas et al. 2022, Breyer et al. 2023). These studies have begun to describe the physiological and morphological characteristics of marine yeast growth *in vitro*, but trait-based data remain scarce, particularly responses to nutrient availability and in relation to open ocean ecosystems. To address these gaps in understanding of planktonic marine yeast ecophysiology, this study quantifies the growth of open ocean yeast isolates under variable nutrient conditions in laboratory culture. Five strains isolated from the North Atlantic as part of the Marine Biological Association Fungal Culture Collection, were assessed with a phenotype microarray for the ability to use a diversity of nitrogen-containing compounds as the primary nitrogen source. One model strain, *Naganishia diffluens* MBA_F0213, was here exposed to nitrogen concentrations spanning several orders of magnitude in three environmentally relevant chemical forms: ammonium, nitrate, and urea. Observations of cell density, growth substrate uptake, and cell morphology are used to describe *N. diffluens* MBA_F0213 responses to nitrogen form and concentration. Meta-analysis of the reference DNA barcode in public databases is used to explore the marine biogeographic distribution of the genus *Naganishia*, to contextualize the nitrogen dynamics observed *in vitro* with *N. diffluens* MBA_F0213 cultures.

## Methods

### Cultures and growth media

Five yeast and yeast-like cultures were selected from the Marine Biological Association Fungal Culture Collection (Table 1). All strains were isolated from the North Atlantic Ocean at the Porcupine Abyssal Plain Sustained Observatory (PAP-SO) (Supplementary material) in June 2019. Cultures were maintained on Wickerham’s yeast malt medium (WYM) or potato dextrose medium (PDM) agar plates at either 10°C or 15°C depending on the isolation depth (Table 1).

**Table 1.** Yeast strains used in this study. Marine Biological Association Fungal Culture Collection strain codes with species identification, water depth of isolation, agar media formulation of isolation/maintenance, and temperature of culture maintenance.

Strain	Species	Isolation depth (m)	Media	Temperature (°C)
MBA_F0175	*Holtermanniella festucosa*	1000	WYM	10
MBA_F0181	*Aureobasidium pullulans*	500	PDM	10
MBA_F0213	*Naganishia diffluens*	8	PDM	15
MBA_F0294	*Rhodotorula mucilaginosa*	30	PDM	15
MBA_F0295	*Sporobolomyces roseus*	30	PDM	15

For all experiments, liquid cultures were prepared in seawater f/2 medium (Guillard 1975) with minor modifications as follows. Glucose was added to filtered natural seawater at 1.8 g l^−1^ and autoclaved. Sodium phosphate (NaH_2_PO_4_·H_2_O), trace metals, and vitamin solutions were filter-sterilized (0.22 µm) and added to the autoclaved seawater at concentrations in line with the standard f/2 protocol (Guillard 1975). Chloramphenicol was added at 0.2 g l^−1^. The form and concentration of nitrogen substrates added varied depending on experimental treatment (see below). As natural seawater forms the base for this growth medium, background dissolved nutrient concentrations depend on the environmental source. Autoclaved filtered natural seawater as used here was shown to contain ∼3.4 µmol l^−1^ nitrate and ∼0.9 µmol l^−1^ ammonium (Supplementary material). Urea concentration could not be determined in this analysis. Any nitrogen substrate additions to the f/2 medium are therefore considered nitrogen enrichments to these background concentrations.

### Diversity of nitrogen substrate use

**Figure 1.**
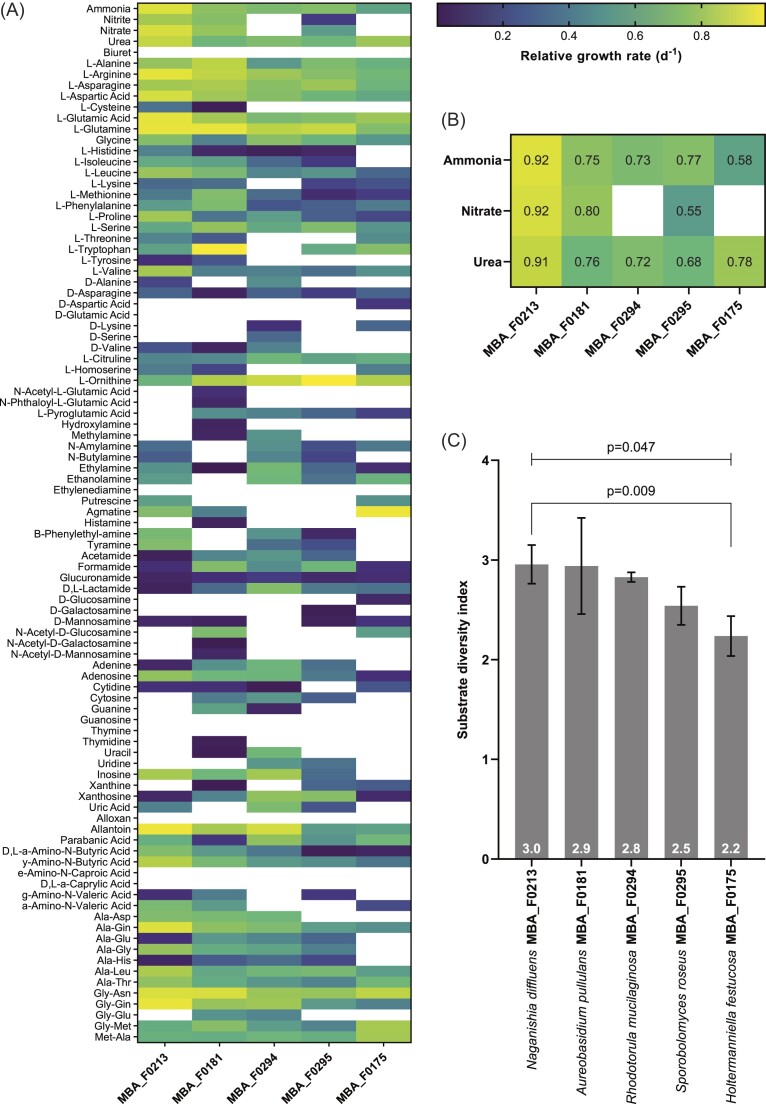
(A) Growth rates of five marine yeast and yeast-like fungal strains on 95 nitrogen sources, normalized to the maximum growth rate achieved by each strain. Colour indicates relative growth rate. White space indicates no significant growth above the non-nitrogen-enriched f/2 medium control. Nitrogen enrichments are between 1 and 5 mM, except amino acids, purines, and pyrimidines, which are included at 20–200 µM (BIOLOG, personal communication). (B) Growth rates with enrichment of ammonia, nitrate, or urea (expanded from A). (C) Substrate diversity index based on the number of nitrogen substrates yielding positive growth rates. Plotted values and labels indicate the mean (*n* = 3). Error bars indicate the standard deviation.

The diversity of nitrogen substrate use was screened using 96-well culture plates containing 95 different nitrogen sources and a negative control (BIOLOG PM3B Phenotype MicroArray). This commercially produced assay is designed to test for microbial utilization of a diverse array of nitrogen sources, including ammonia, nitrate and urea, as well as various amino acids, amines and nucleobases (Fig. 1A). Nitrogen enrichments are between 1 and 5 mM, except amino acids, purines, and pyrimidines, which are included at 20–200 µM (BIOLOG, personal communication). Late exponential phase cultures were washed and resuspended in modified f/2 medium (see above) with no nitrogen enrichment. Washed cell suspensions were diluted to ∼10^4^ cells ml^−1^, added at 100 µl per well and incubated at 15°C for 4 days. Triplicate plates were inoculated for each strain. Optical density at 600 nm (OD_600_) was measured daily using a microplate reader (CLARIOstar) and used to calculate growth rates. Relative growth rate is the growth rate on each substrate normalized by the maximum growth rate of that strain on any substrate as measured in the BIOLOG microarray (Supplementary material). A substrate diversity index was used to quantify the range of substrates used by each strain based on the number of substrates yielding positive growth rates (Thomas et al. 2022) (Supplementary material).

### Nitrogen concentration range

Growth responses of *N. diffluens* MBA_F0213 to ammonium ((NH_4_)_2_SO_4_), nitrate (NaNO_3_), or urea (CO(NH_2_)_2_) were assessed in batch culture experiments with initial media enrichments of 0.1, 1, 10, 100, 1000, or 9000 µmol l^−1^ N. The dissolved carbon source of 1.8 g l^−1^ glucose corresponds to 10 mmol l^−1^ glucose or 60 mmol l^−1^ carbon. The uppermost N addition of 9 mmol l^−1^ was derived from this carbon concentration using a C:N ratio of ∼108:16 representing the average composition of marine organic matter (Redfield 1934). The lowest initial N concentration tested here (with 0.1 µmol l^−1^ added N) is ∼4.6 µmol l^−1^ N, accounting for dissolved inorganic nitrogen present in the natural seawater (Supplementary material). The lower [N] range, therefore encompasses nitrogen concentrations relevant in the open ocean e.g. surface [NO_3_^−^] < 10 µmol l^−1^ at PAP-SO (Hartman et al. 2015).

Liquid batch cultures of 40 ml in vented polystyrene flasks (Sarstedt T-25) were incubated at 15°C, orbitally shaken at 100 r/m and lit by a 14:10 h light–dark cycle. *Naganishia diffluens* MBA_F0213 cells were acclimated to each treatment condition for at least three subcultures (each ∼5 days) prior to experiments. Fresh growth media were inoculated in triplicate with late-exponential phase cultures to a cell density of ∼10^4^ cells ml^−1^ to initiate experiments. Experiments were terminated when incubations reached stationary phase, indicated by no significant difference in cell density between consecutive daily time points.

Cell density was monitored with daily cell counts on an Improved Neubauer hemocytometer (Hawksley) and Leica DM1000 LED microscope. Specific growth rate values were obtained in triplicate for each treatment. A modified logistic model was fit to log-transformed cell density data (Zwietering et al. 1990) or where the data fit the model poorly, estimated as the maximum gradient between individual time points in the exponential phase (Supplementary material). A growth rate value of 0 was assigned when there was no significant difference in means (*n* = 3) between the final and initial cell densities.

Media subsamples of 1 ml were taken daily, centrifuged at 16 000 × *g* for 5 min and the supernatant stored at −20°C. d-glucose concentrations were determined using a glucose oxidase assay (Invitrogen Amplex Red A222189). Glucose uptake was calculated as the difference between the final and initial media glucose concentrations.

At each time point, at least 10 cells per sample were imaged using Leica LAS EZ software. Cell morphometry was quantified using image analysis software (ImageJ). Ellipses were manually fit to 10 cells per sample, outputting mean measurements of the major and minor elliptical axes. Cell dimensions were used to estimate cell eccentricity [a measure of ellipsoidal shape elongation i.e. how much the cell deviates from being circular, which could have a role in the efficiency of nutrient uptake (Yan et al. 2021)] and cell surface area to volume ratio (equations in Supplementary material).

### Naganishia DNA barcode meta-analysis

Strain MBA_F0213 was previously identified as the basidiomycete yeast *N. diffluens* (order *Filobasidiales*) by DNA sequencing of the ITS region. According to an integrated phylogenetic classification (Liu et al. 2015), all type strains of species within the *Filobasidiales* were queried against the NCBI nucleotide database (Sayers et al. 2022) to identify all available 18S rRNA encoding gene reference sequences. A phylogenetic tree was generated by pairwise 18S sequence alignments using the NCBI BLAST Tree View tool (Altschul et al. 1990). AliView software was used to generate a MAFFT alignment of the reference sequences, from which the 18S rRNA gene hypervariable V9 regions were identified (Supplementary material). The V9 regions were compared to assess the taxonomic resolution of potential metabarcode sequences from the *Filobasidiales* in the Tara Oceans eukaryote 18S V9 database (De Vargas et al. 2015). The 18S rRNA gene reference sequence NG_062944.1 from *N. diffluens* CBS 160 type material was queried against the Tara Oceans database using the Ocean Barcode Atlas web portal (Vernette et al. 2021). Operational taxonomic units (OTUs) with >97% identity to reference sequence NG_062944.1 were selected to represent *Naganishia* at the genus level. The marine geographic distribution of *Naganishia* OTUs was analysed using the Ocean Barcode Atlas. Relative abundance data represent OTU counts as a fraction of total metabarcode reads in each sample.

### Statistical analyses and data availability

Growth data were processed and visualized using GraphPad Prism 9.5.1. DNA sequences were aligned and visualized using AliView. Statistical analyses were carried out in Minitab 20.3. The Kruskal–Wallis test was used to test for differences between treatments with significance reported at *P* < .05 unless otherwise stated. Graph error bars represent the standard deviation. All raw data used in this study are available as a spreadsheet file (Supplementary material).

## Results

### Nitrogen substrate use diversity

The five marine yeast and yeast-like fungal isolates can grow using a range of nitrogen-containing compounds as the primary nitrogen source (Fig. 1A). All strains grew with enriched ammonia and urea, but only *N. diffluens* MBA_F0213, *Aureobasidium pullulans* MBA_F0181 and *Sporobolomyces roseus* MBA_F0295 grew with enriched nitrate (Fig. 1B). There were strain-specific differences in nitrogen substrate use diversity (*P* = .047) (Fig. 1C). *Naganishia diffluens* MBA_F0213 grew on the broadest diversity of nitrogen substrates, yielding the highest substrate diversity index (2.96 ± 0.19) of the five strains.

### Physiological responses of *N. diffluens* to nitrogen substrate and concentration

**Figure 2.**
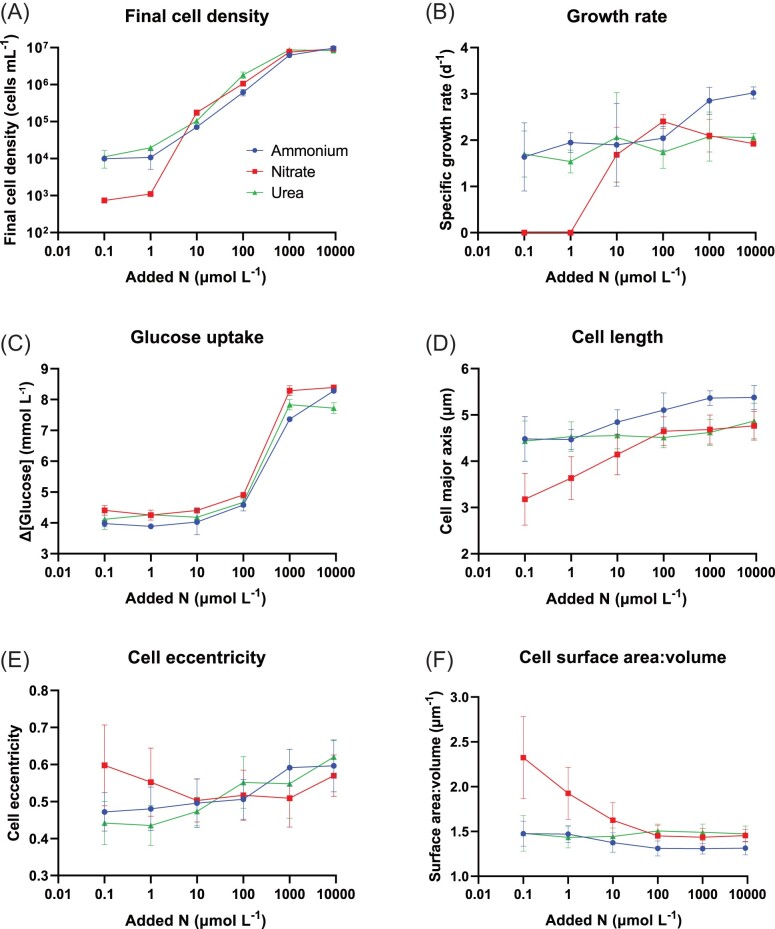
Physiological and morphological responses of *N. diffluens* MBA_F0213 to nitrogen enrichment in three different forms: ammonium, nitrate and urea. (A) Cell density at the final time point. (B) Specific growth rate based on cell density measurements. (C) Total glucose uptake from culture media. (D) Cell size (major elliptical axis). (E) Cell shape (elliptical eccentricity). (F) Estimated cell surface area to volume ratio based on measurements of major and minor elliptical axes. Morphological data (D)–(F) include measurements from all time points before stationary phase (5–9 days). Plotted values indicate the mean (*n* = 3 for A–C, *n* ≥15 for D–F). Error bars indicate standard deviation.

The final cell density (i.e. yield) of *N. diffluens* MBA_F0213 increased with increasing nitrogen enrichment between 0.1 and 9000 µmol l^−1^ for all three nitrogen substrates (*P* ≤ .007) (Fig. 2A) and peaked at 9.67 ± 0.64 × 10^6^ cells ml^−1^ (9000 µmol l^−1^ added ammonium treatment). Below 10 µmol l^−1^ added N, final cell density was lower on nitrate than on urea (*P* = .027), suggesting that *N. diffluens* MBA_F0213 population size is controlled by nitrogen speciation under low nitrogen conditions.

Specific growth rate increased between 1 and 100 µmol l^−1^ added nitrate (*P* = .004) but was unaffected by the concentration of ammonium or urea (Fig. 2B). Growth rates were below detection at 0.1–1 µmol l^−1^ added nitrate (no significant difference between final and initial cell densities). At 9000 µmol l^−1^ added N, specific growth rate was higher on ammonium (3.02 ± 0.13 d^−1^) than on nitrate (1.92 ± 0.02 d^−1^) (*P* = .007), suggesting *N. diffluens* MBA_F0213 grows more efficiently on reduced inorganic nitrogen than on oxidized inorganic nitrogen at high concentrations.

Total glucose uptake increased with increasing nitrogen enrichment for all three nitrogen substrates between 10 and 1000 µmol l^−1^ N (*P* ≤ .009) (Fig. 2C) and peaked at 8.39 ± 0.05 mmol l^−1^ (9000 µmol l^−1^ added nitrate treatment).

### Morphological responses of *N. diffluens* to nitrogen substrate and concentration

Cell length increased with increasing nitrogen enrichment between 0.1–100 µmol l^−1^ nitrate (*P* < .001), 1–100 µmol l^−1^ ammonium (*P* < .001), and 100–9000 µmol l^−1^ urea-N (*P* = .002) (Fig. 2D). Below 10 µmol l^−1^ added N, cell length was lower on nitrate than on ammonium or urea (*P* < .001). Above 10 µmol l^−1^ added N, cell length was higher on ammonium than on nitrate or urea (*P* ≤ .002). Cell length varied from a maximum of 5.38 ± 0.26 µm (9000 µmol l^−1^ added ammonium) down to 3.18 ± 0.56 µm (0.1 µmol l^−1^ added nitrate), suggesting that *N. diffluens* MBA_F0213 cells reduce in length by up to ∼40% in response to nitrogen scarcity.

Cell eccentricity (a measure of ellipsoidal shape elongation) increased with increasing nitrogen enrichment between 100–1000 µmol l^−1^ ammonium (*P* = .002), 10–9000 µmol l^−1^ nitrate (*P* = .004) and 10–9000 µmol l^−1^ urea-N (*P* < .001) (Fig. 2E), ranging from 0.435 ± 0.054 (1 µmol l^−1^ added urea-N) to 0.620 ± 0.044 (9000 µmol l^−1^ added urea-N). The reverse trend was observed under low nitrate enrichment with cell eccentricity decreasing between 1–10 µmol l^−1^ added nitrate (*P* = .008). Below 10 µmol l^−1^ N enrichment, cells were more eccentric on nitrate than on ammonium or urea (*P* ≤ .023), suggesting *N. diffluens* MBA_F0213 changes cell shape, as well as length (see above), under variable nitrogen conditions.

Cell surface area to volume ratio decreased with increasing nitrogen enrichment between 1 and 100 µmol l^−1^ nitrate (*P* < .001) and 1–100 µmol l^−1^ ammonium (*P* < .001), but increased slightly between 1 and 100 µmol l^−1^ added urea (*P* = .004) (Fig. 2F). The minimum cell surface area to volume ratio of 1.31 ± 0.06 µm^−1^, observed in the 1000 µmol l^−1^ added ammonium treatment, was lower than for nitrate or urea at the same concentration (*P* < .001). At 0.1–10 µmol l^−1^ added N, cell surface area to volume was higher on nitrate than on ammonium or urea (*P* ≤ .003), indicating that the changes in cell length and shape under low nitrate enrichment (cells became smaller and more elongated) had the combined result of increasing cell surface area to volume. In combination with the physiological data (i.e. no growth detected below 10 µmol l^−1^ added nitrate), this suggests a morphological stress response to nitrogen limitation in *N. diffluens* MBA_F0213.

Growth rate, cell length and cell surface area to volume are all affected by nitrogen enrichment in the form of nitrate, which could be explained by allometric relationships between physiological processes and cell size in *N. diffluens* MBA_F0213, whereby decreasing size would be expected to increase nutrient affinity. In contrast with nitrate, urea enrichment does not affect growth rate, nor does it result in decreasing cell surface area to volume, despite increasing cell length. The changes in cell shape but lack of growth rate response to urea or ammonium enrichment suggest that *N. diffluens* MBA_F0213 adapts cell morphology while maintaining physiological rates across variable nutrient conditions in some cases.

### Distribution of *Naganishia* in the global ocean

**Figure 3.**
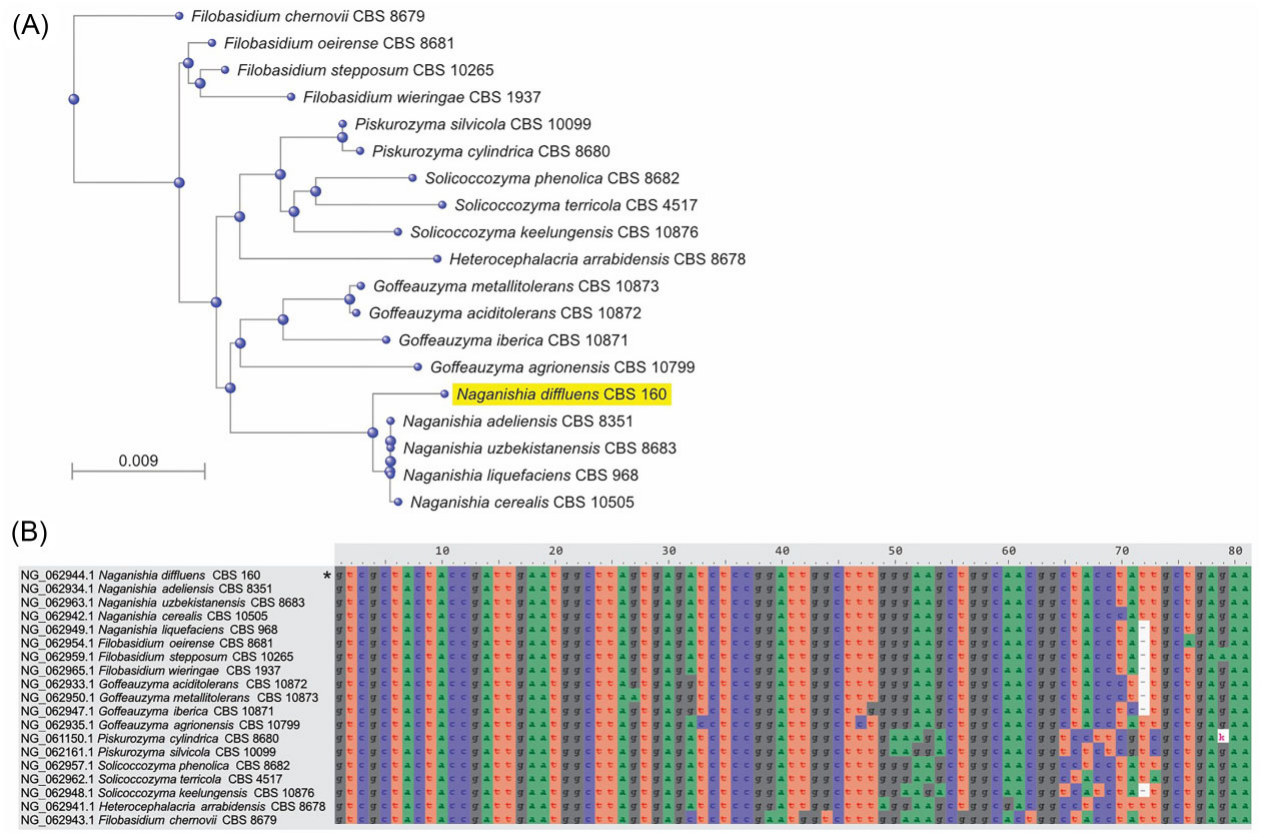
(A) Phylogenetic tree of the 19 species within order Filobasidiales, which have a type strain 18S rRNA encoding gene reference sequence available on NCBI. Tree generated using pairwise 18S sequence alignments on BLAST Tree View tool, NCBI. (B) Visualization of partial V9 regions of MAFFT aligned 18S rRNA encoding gene reference sequences of Filobasidiales.

**Figure 4.**
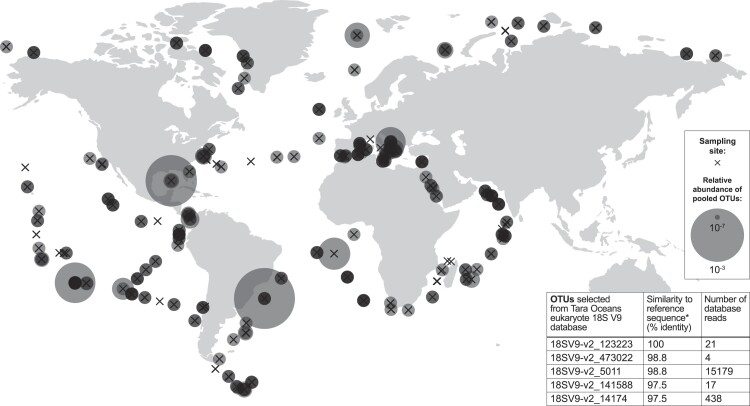
Distribution of *Naganishia* operational taxonomic units (OTUs) in the global ocean. OTUs from the Tara Oceans eukaryote 18S V9 database selected due to >97% (genus level) similarity to *N. diffluens* (inset table). Circle area is proportional to relative OTU abundance, i.e. pooled counts of the five selected OTUs, as a fraction of total metabarcode reads. Crosses mark sampling sites. Plotted using Ocean Barcode Atlas web tool.

There are 19 species within order *Filobasidiales* with a type strain 18S rRNA encoding gene reference sequence available on NCBI, including five from the genus *Naganishia* (Fig. 3A). Using the V9 region of the 18S rRNA gene type strain reference sequence alone, *N. diffluens* is not distinguishable from two other *Naganishia* species (*N. adeliensis* and *N. uzbekistanensis*) but differs from all other *Filobasidiales* tested (Fig. 3B). Querying the Tara Oceans eukaryote 18S V9 metabarcode database yielded five OTUs with >97% (genus level) identity to the *N. diffluens* reference sequence, totalling 15 659 reads (Fig. 4, inset). Pooled relative abundances of the five selected OTUs demonstrate that genus *Naganishia* is widely distributed in the global ocean, including at open ocean locations (Fig. 4).

## Discussion

Here we have shown that marine yeasts can use a broad diversity of nitrogen substrates, that varies between strains, possibly due to differing enzymatic capabilities in different species. It is important to reiterate that the concentrations used in the BIOLOG assay (Fig. 1) are higher than observed in the open ocean and, therefore we are not considering any concentration dependent factors in this part of our study. Some of the nitrogen substrates in the BIOLOG assay could also have potential toxic effects on some yeasts, such as nitrite (Kubisi et al. 1996). Yeasts use membrane transporters (permeases) to take up nitrogenous compounds and additional enzymes to convert different molecules into a form that is readily assimilated, commonly ammonium (Villers et al. 2017). All marine yeasts in this study were able to grow with ammonium or urea as the primary nitrogen source. Although urea (CO(NH_2_)_2_) is a reduced form of nitrogen, cells must catalyse the hydrolytic cleavage of urea to release ammonium. Some ascomycete yeasts and unicellular green algae use ATP-dependent urea amidolyase to degrade urea, while other fungi, microalgae, and bacteria use nickel-containing ureases (Bekheet and Syrett 1977, Navarathna et al. 2010). The only ascomycete yeast-like fungus in this study, *A. pullulans* MBA_F0181, has been shown to produce urease (Federici 1982), while the four other strains are basidiomycetes, which universally possess genes encoding urease and an associated nickel transporter (Zhang et al. 2009, Navarathna et al. 2010). Marine yeast assimilation of urea using urease would impose a nickel dependency, which could potentially become nickel–nitrogen colimitation, as demonstrated in cultures of the marine diatom *Thalassiosira weissflogii* (Price and Morel 1991) and marine cyanobacterium *Synechococcus* (Dupont et al. 2008), as well as natural phytoplankton communities in offshore Pacific surface waters (Dupont et al. 2010). Urea-utilizing marine yeasts may therefore contribute to the biological drawdown of nickel, which results in characteristic nutrient depth profiles of marine nickel concentrations (Sclater et al. 1976).

To convert nitrate to ammonium, some yeasts use nitrate and nitrite reductases (Siverio 2002), with an analogous pathway in photosynthetic eukaryotes (Sanz-Luque et al. 2015). Here, we found that marine isolates *N. diffluens* MBA_F0213, *A. pullulans* MBA_F0181, and *S. roseus* MBA_F0295 are able to grow with nitrate as the primary nitrogen source, in agreement with previous studies on nonmarine isolates of these species (Ali and Hipkin 1985, Zheng et al. 2008, Fotedar et al. 2018). *Rhodotorula mucilaginosa* MBA_F0294 and *Holtermanniella festucosa* MBA_F0175 did not grow with enriched nitrate, in agreement with evidence for an incomplete nitrate assimilation pathway in *R. mucilaginosa* (Sen et al. 2019) and the absence of nitrate assimilation throughout genus *Holtermanniella* (Wuczkowski et al. 2011). *Sporobolomyces* and *Rhodotorula* (phylum Basidiomycota, subdivision Pucciniomycotina) were both identified by 18S rRNA gene tag sequencing during a coastal spring phytoplankton bloom, with cell counts and biomass of Pucciniomycotina significantly positively correlating with nitrate concentration (Priest et al. 2021), suggesting related marine yeast growth responds to nitrate availability in pelagic marine environments.

Ammonium is the energetically favoured nitrogen source within cells given its direct synthesis into glutamate and subsequently macromolecules (Sanz-Luque et al. 2015, Villers et al. 2017), but dissolved ammonium is typically a scarce nutrient in marine surface waters due to rapid microbial assimilation (Glibert and Goldman 1981). The ability to use nitrate could give some yeasts a competitive advantage over microbes reliant on reduced nitrogen, particularly in nitrate-rich marine waters found commonly at depth and sporadically in the surface open ocean due to upwelling or vertical mixing events. This potential benefit could be amplified where high nitrate coincides with an abundance of bioavailable organic carbon, such as the biogeochemically important phytoplankton-derived polysaccharide laminarin (Becker et al. 2020), which fungal plankton including basidiomycete yeasts assimilate in coastal ecosystems (Cunliffe et al. 2017). Initial evidence for nitrate-supported marine yeast growth comes from Helgoland Roads in the German Bight, where peak yeast abundance (3.3 × 10^5^ cells l^−1^) coincided with relatively high nitrate (23.02 µmol l^−1^) compared to ammonia (1.26 µmol l^−1^) during a spring phytoplankton bloom dominated by pennate diatoms (Priest et al. 2021). At concentrations below 10 µmol l^−1^ nitrate [0.1 and 1 µmol l^−1^ nitrate enrichments equate to media concentrations of ∼3.5 µmol l^−1^ and ∼4.4 µmol l^−1^ nitrate, respectively (Supplementary material)], which are typical in the North Atlantic Ocean (Hartman et al. 2015), the lack of detectable population growth in *N. diffluens* MBA_F0213 suggests a possible survival trade-off between cellular energy investments in nitrate uptake versus reproduction by cell division.

It should be noted that the upper range of ammonium, nitrate and urea concentrations explored in the *N. diffluens* MBA_F0213 culture experiments (100–9000 µmol l^−1^ N) are unrealistic in the marine environmental context. Also, because we used natural seawater containing ∼3.4 µmol l^−1^ nitrate and ∼0.9 µmol l^−1^ ammonium, nitrogen substrate additions are enrichments. Across the euphotic Atlantic Ocean, peak concentrations of 5.2 µmol l^−1^ ammonium, 33 µmol l^−1^ nitrate, and 1.72 µmol l^−1^ urea have been observed (Rees et al. 2006, Painter et al. 2008). The lowest media glucose concentration across the experiment was still >1 mmol l^−1^, corresponding to >6 mmol l^−1^ DOC, in excess of typical environmental DOC concentrations. Annual mean DOC in the Western English Channel off Plymouth is ∼0.07 µmol l^−1^ (Hochman et al. 1995), which is ∼10^5^ times lower than the most glucose-depleted media at the end of this experiment. Despite low average concentrations, nutrient availability in the marine water column is heterogeneous at the microscale (Stocker 2012), with nitrogen substrate concentrations sometimes orders of magnitude higher around marine aggregates (Shanks and Trent 1979). Therefore, the responses of *N. diffluens* MBA_F0213 to high nitrogen could bear some relevance to marine yeast ecophysiology at the microscale.

Pelagic osmotrophs face a trait trade-off between nutrient uptake and predator defence, with smaller cells having a competitive advantage in diffusive nutrient uptake, while larger cells avoid predation by small, abundant, and fast-growing zooplankton (e.g. Acevedo-Trejos et al. 2015). The smaller cell sizes of *N. diffluens* MBA_F0213 under low nitrogen enrichment could be explained by size-dependent nutrient acquisition in marine yeasts. Applying allometric parameters empirically determined in phytoplankton by Edwards et al. (2012), a 40% decrease in cell length (as observed in *N.diffluens* MBA_F0213) would increase biomass-scaled nitrogen affinity 2.6-fold. Under high nutrient conditions, larger cell sizes could reduce grazing pressure in pelagic ecosystems (Lürling 2021). Larger marine yeasts may also have greater capacity for intracellular storage of nonlimiting resources, as has been shown in heterotrophic bacteria, with excess glucose causing carbon-rich cell inclusions (Thingstad et al. 2005). Diverse microbial eukaryotes are capable of intracellular nitrogen storage (Kamp et al. 2015) including yeasts, which sustain growth using internally accumulated nitrogen when external nitrogen is depleted (Gutiérrez et al. 2016). Increased internal storage of glucose or nitrogen could explain why *N. diffluens* MBA_F0213 increases cell size with increasing nitrogen concentrations but this would require validation through macromolecular analysis.

Cell surface area to volume ratio (a product of cell size and shape), affects phytoplankton nutrient uptake potential and sinking (Lewis Jr 1976). Yeasts have low surface area to volume ratios relative to filamentous fungi, reducing contact with the extracellular environment and potentially affecting yeast nutrient uptake and sedimentation rates in aquatic ecosystems (El Baidouri et al. 2021). By increasing cell surface area to volume under low nitrogen availability, *N. diffluens* MBA_F0213 could theoretically achieve more efficient dissolved nutrient uptake per unit cell volume, as described in phytoplankton ecology (Edwards et al. 2012). Again, it is important to state the higher nitrate concentrations used in this study when the surface area-to-volume ratio stops changing (i.e. > 100 µmol l^−1^) are unrealistic in the marine environmental context. There was no response in the surface area-to-volume ratio of *N. diffluens* MBA_F0213 under increasing ammonium and urea concentrations. It is possible that neither of these two reduced dissolved nitrogen species are common sources of nutrient for the yeast in the open ocean, perhaps because other plankton such as bacteria are better adapted for ammonium and urea assimilation. Future studies could consider nutrient uptake competition experiments between marine yeasts and bacteria.

According to a size-based classification of all pelagic marine life (Andersen et al. 2016), at ∼3–6 µm cell length, *N. diffluens* MBA_F0213 falls within the characteristic size range of unicellular phototrophs. Considering trophic strategies, marine yeasts are more closely comparable to osmo-heterotrophic bacteria, which also use organic carbon but are typically an order of magnitude smaller in size (Andersen et al. 2016). The common trophic strategies and distinct cell sizes of marine yeasts and heterotrophic bacteria could have important implications for understanding the fate of organic carbon in marine ecosystems. If marine yeasts are truly functionally distinct from other plankton, they likely mediate flows of energy and nutrients not currently represented in ecological models, given that cell size and trophic mode are key traits used to define plankton functional groups (Barton et al. 2013).

Based on Tara Oceans sampling locations, the yeast genus *Naganishia* has a wide marine distribution, suggesting species within this genus are adapted for survival in a broad spectrum of oceanographic conditions. This evidence adds weight to the idea that the morphology, reproduction, and dispersal strategies of unicellular budding yeasts make them well suited to a planktonic mode of life (El Baidouri et al. 2021). Sampling of *Naganishia*-assigned OTUs at low-latitude open ocean locations where surface nitrogen concentrations commonly limit microbial productivity (Moore et al. 2013), suggests *Naganishia* species encounter low nitrogen availability *in natura*, but OTU counts alone do not allow the environmental trait variability of this genus to be explored. Given its isolation from the surface North Atlantic, the adaptive physiological and morphological responses of *N. diffluens* MBA_F0213 to low nitrogen availability *in vitro* provide insight into the possible mechanisms of yeast survival in surface ocean nutrient conditions. We postulate that marine yeast traits, and therefore ecological functions, vary across environmental gradients such as dissolved nitrogen availability in the open ocean.

To develop a functional understanding of marine yeast ecology, future research will need to incorporate observations of cell physiology and morphology *in natura*, to complement culture- and DNA-based approaches. Quantification of yeast population/biomass dynamics in open ocean environments, as previously demonstrated for a coastal ecosystem (Priest et al. 2021), would improve the scalability of yeast trait observations to pelagic ecosystems more broadly. We advocate for the application of trait-based approaches to characterizing marine-occurring yeasts and marine fungi in general, as a way to unify principles from fungal and plankton ecology and to advance our understanding of the structure and function of pelagic ecosystems.

## Supplementary Material

fiae053_Supplemental_File
