# Supplementary material for: Physiological and morphological plasticity in response to nitrogen availability of a yeast widely distributed in the open ocean
Source: FEMS Microbiol Ecol. 2024 Apr 10;100(5):fiae053. doi: 10.1093/femsec/fiae053 (PMC11062419; doi:10.1093/femsec/fiae053)
Supplement: fiae053_Supplemental_File [file fiae053_supplemental_file.docx]

**Physiological and morphological plasticity in response to nitrogen availability of a yeast widely distributed in the open ocean**

**Supplementary materials**

Porcupine Abyssal Plain Sustained Observatory (PAP-SO)


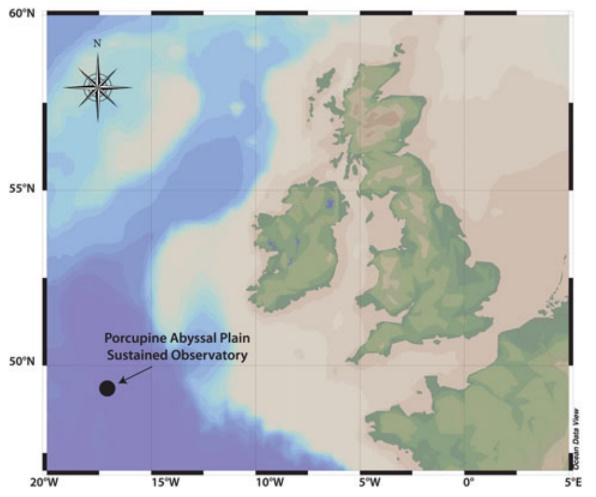


Location of the Porcupine Abyssal Plain Sustained Observatory (48°50′N 16°30′W) in the North Atlantic Ocean (Kazanidis et al. 2014).

Natural seawater nutrient analysis

Table below shows nutrient contents of autoclaved filtered natural seawater used as base for growth media in culture experiments. Seawater collected from L4 Station, approximately 16km southwest of Plymouth in the Western English Channel. Analysed by Malcolm Woodward at Plymouth Marine Laboratory. Mean values stated ± standard deviation (n=5).

| Nutrient | Concentration (µM) |
| --- | --- |
| Nitrite | 0.18 ± 0.00 |
| Nitrate + nitrite | 3.57 ± 0.05 |
| Ammonium | 0.89 ± 0.57 |
| Silicate | 146.51 ± 0.24 |
| Phosphate | 0.52 ± 0.01 |

Equations

Growth rate:

$$\mu=\frac{\ln(N_{t}/N_{0})}{t-t_{0}}$$

Based on the change in cell density *N* between consecutive time points *t_0_* and *t*.

Relative growth rate:

$$\mu_{r}=\frac{\mu}{\mu_{\max}}$$

Where the growth rate on each substrate *µ* is normalised by the maximum growth rate of that strain on any substrate *µ_max_* as measured in the BIOLOG microarray.

Modified logistic model:

$$\ln\left( \frac{N_{t}}{N_{0}} \right)=\frac{A}{\left\{ 1+exp\left[ \frac{4\mu_{s}}{A}\left( \lambda-t \right)+2 \right] \right\}}$$

Where the specific growth rate *µ_s_* is the maximum gradient of the growth curve which has a lag time *λ* and a maximum cell density *e^A^* (Zwietering et al. 1990; Ward et al. 2017).

Substrate diversity index:

$$H=-\sum_{i=1}^{N} p_{i}(\ln p_{i})$$

Where *H* is the Shannon diversity index, *N* is the number of substrates yielding a positive growth rate and *p_i_* is the proportion of total growth by each substrate *i* (Thomas et al. 2022).

Glucose uptake:

$$\Delta[Glucose]=\left[ Glucose \right]_{final}-{[Glucose]}_{initial}$$

Cell morphometry:

$$E= \sqrt{1-({b^{2}}/{a^{2}})}$$

$$SA\approx4\pi\left( \frac{\left( ab \right)^{1.6}+\left( ab \right)^{1.6}+\left( bb \right)^{1.6}}{3} \right)^{1/{1.6}}$$

$$V\approx(4/3)\pi ab^{2}$$

Where 2*a* and 2*b* are major and minor cell axes respectively, *E* is cell eccentricity, *SA* is an approximation of cell surface area and *V* is an approximation of cell volume.

*Filobasidiales* 18S rRNA sequences – V9 region identification

V9 hypervariable regions were identified within 18S rRNA encoding gene reference sequences using the universal 1389F primer sequence (Amaral-Zettler et al. 2009). Given that the eukaryotic reverse primer 1510R was not found in the selected 18S sequences and that only 81 base pairs followed the forward primer (87 – 186 bp were found in a reference V9 database (Amaral-Zettler et al. 2009)), the V9 region sequences presented in this study are assumed to be incomplete.

**References**

Amaral-Zettler, L.A., McCliment, E.A., Ducklow, H.W. and Huse, S.M., 2009. A method for studying protistan diversity using massively parallel sequencing of V9 hypervariable regions of small-subunit ribosomal RNA genes. *PloS one*, *4*(7), p.e6372.

Kazanidis, G., Tyler, P.A. and Billett, D.S., 2014. On the reproduction of the simultaneous hermaphrodite Paroriza prouhoi (Holothuroidea: Synallactidae) in the Porcupine Abyssal Plain, north-east Atlantic. *Journal of the Marine Biological Association of the United Kingdom*, *94*(4), pp.847-856.

Thomas, S., Lengger, S.K., Bird, K.E., Allen, R. and Cunliffe, M., 2022. Macromolecular composition and substrate range of three marine fungi across major cell types. *FEMS Microbes*, *3*.

Ward, B.A., Marañón, E., Sauterey, B., Rault, J. and Claessen, D., 2017. The size dependence of phytoplankton growth rates: A trade-off between nutrient uptake and metabolism. *The American Naturalist*, *189*(2), pp.170-177.

Zwietering, M.H., Jongenburger, I., Rombouts, F.M. and Van't Riet, K.J.A.E.M., 1990. Modeling of the bacterial growth curve. *Applied and environmental microbiology*, *56*(6), pp.1875-1881.
